# A Novel Tolerogenic Antibody Targeting Disulfide-Modified Autoantigen Effectively Prevents Type 1 Diabetes in NOD Mice

**DOI:** 10.3389/fimmu.2022.877022

**Published:** 2022-08-11

**Authors:** Wei Li, Yan Zhang, Ronghui Li, Yang Wang, Lan Chen, Shaodong Dai

**Affiliations:** ^1^ Skaggs School of Pharmacy and Pharmaceutical Sciences, University of Colorado Anschutz Medical Campus, Aurora, CO, United States; ^2^ National Health Commission (NHC) Key Laboratory of Pulmonary Immune-Related Diseases, Guizhou Provincial People’s Hospital, Guiyang, China; ^3^ Department of Immunology and Microbiology, University of Colorado School of Medicine, Aurora, CO, United States

**Keywords:** type 1 diabetes, monoclonal antibody, IAPP, T cells, redox regulation, MHC

## Abstract

Increasing evidence suggested that the islet amyloid polypeptide (IAPP) is an essential autoantigen in the pathogenesis of type 1 diabetes (T1D) in humans and non-obese diabetic (NOD) mice. A unique disulfide containing IAPP-derived peptide KS20 is one of the highly diabetogenic peptides in NOD mice. The KS20-reactive T cells, including prototypic pathogenic BDC5.2.9, accumulate in the pancreas of prediabetic and diabetic mice and contribute to disease development. We generated a monoclonal antibody (LD96.24) that interacts with IA^g7^-KS20 complexes with high affinity and specificity. LD96.24 recognized the IA^g7^-KS20 disulfide loop and blocked the interaction between IA^g7^-KS20 tetramers and cognate T cells but not other autoantigen-reactive T cells. The *in vivo* LD96.24 studies, at either early or late stages, drastically induced tolerance and delayed the onset of T1D disease in NOD mice by reducing the infiltration of not only IAPP-specific T cells but also chromogranin A and insulin-specific T cells in the pancreas, together with B cells and dendritic cells. LD96.24 can also significantly increase the ratio of Foxp3^+^ regulatory T cells with Interferon-gamma-secreting effector T cells. Our data suggested the important role of disulfide-modified peptides in the development of T1D. Targeting the complexes of Major histocompatibility complex (MHC)/disulfide modified antigens would influence the thiol redox balance and could be a novel immunotherapy for T1D.

## Introduction

CD4^+^ T cells recognize self-antigens from β cells and lead to the destruction of these insulin (Ins)-producing cells. Autoreactive T-cell receptor (TCR), MHC class II, and peptide trimolecular complexes play an important roles in the pathogenic islet-specific CD4^+^ T-cell activation in type 1 diabetes (T1D). The highest T1D genetic risk factor is a single polymorphism in the β chain of MHC class II molecule, H2-IA^g7^ in mice and human leukocyte antigen-DQ8 in humans within the amino acid for β57, which are responsible for antigen presentation by antigen-presenting cells (APCs) to CD4^+^ T cells (1). Increasing evidence suggests that autoimmunity may be caused by the T-cell recognition of neoantigens, particularly in T1D (2, 3). The post-translational modifications (PTMs) of proteins and their peptide products can lead to autoimmunity. Peptide post-translational processes in neoantigen generation have been implicated in autoimmune diseases, including peptide fusion, citrullination, transglutamination, acetylation, glycosylation, hydroxylation, and phosphorylation (4). Several non-enzymatic processes have also been implicated including neoepitope generation with glycation, oxidation, carbonylation, isoaspartic acid formation, and carbamylation. The T cells specific for these post-translationally modified peptides have been challenging to find, partly because the target peptides cannot be screened by conventional genetic means. Recently, we have suggested that both Ins and ChgA peptides, B:9-23 and WE14, respectively, may be post-translationally modified (5, 6). Islet amyloid forms rapidly in islets transplanted into T1D recipients and likely plays a role in islet graft inflammation and dysfunction (7). IAPP also participates in PTM to form a hybrid Ins peptide (HIP), which is highly antigenic for BDC6.9 and BDC-9.3 T-cell lines, which are prevalent in diabetic NOD mice (8). The KS20 peptide from IAPP is 20 amino acids in length, which is the target of the prototypical diabetogenic CD4^+^ T-cell line, BDC5.2.9 (9). Baker et al. identified two additional IAPP KS20-reactive pathogenic T-cell lines, BDC-5/S3.4 and BDC-9/S3.5 (10). The KS20-reactive T cells can be detected in the pancreas of prediabetic and diabetic NOD mice and contribute to disease development (10). IAPP autoantibodies have also been identified in humans but are not associated specifically with T1D (11, 12). IAPP N-terminus contains a conserved disulfide bridge that was suggested to be important to IAPP aggregation (13). NMR structures showed that the two cysteines of C2 and C7 form a disulfide bridge *in vitro* at the N-terminal of the KS20 peptide (14, 15). We hypothesize that the disulfide bridge may induce a large conformational change of the KS20 peptide and create a major T-cell epitope, forming a neoantigen. The formation of neoantigens is normally a consequence of various types of insults that generate endoplasmic reticulum (ER) stress, reactive oxygen species (ROS), and/or inflammatory cytokines in the affected tissue (4). In T1D, these stresses elicit post-translational process which leads to the generation and release of neoantigens with altered immunogenicity that can be presented to autoreactive T cells, which have escaped negative selection in the thymus.

At present, there is no effective and safe antigen-specific immunologic therapy for T1D. Given the potential of direct inhibition on autoimmunity initiation, there is great interest in developing antibodies targeting primary MHC II-peptide complexes as therapeutic agents. The monoclonal antibodies (mAbs) binding MHC II-peptide can prevent the formation of trimolecular complexes and inhibit T-cell activation. Recently, a high-affinity mAb specific for a gluten peptide bound to MHC II molecules inhibited the activation and proliferation of gluten-specific CD4^+^ T cells *in vitro* and humanized mice (16). Monoclonal antibody (mAbs) against IA^g7^-Ins B:10-23 can modulate the onset of T1D (17, 18). Dahan et al. found that the antibody against DR4/GAD-555-567 complexes significantly inhibited GAD-555-567-specific T-cell responses *in vitro* and *in vivo* (19). Since KS20-reactive T cells are highly pathogenic to the development of T1D (9, 10), we generated an mAb against the disulfide bridge of the KS20 peptide bound to the IA^g7^ molecule with high binding affinity. This mAbs inhibited pathogenic KS20-specific T cells *in vitro* and effectively prevented the development of diabetes *in vivo*.

## Materials and Methods

### Mice

Female NOD/LtJ mice were purchased from the Jackson Laboratories and maintained in the Biological Resource Center at National Jewish Health (Denver, CO, USA). Animal husbandry and experimental procedures were conducted under the protocols approved by the Institutional Animal Care and Use Committee of the National Jewish Health.

### Protein Expression, Purification, and Tetramer Preparation

We constructed the plasmids that the IA^g7^ α- and β-chains were under P10 and PH promoters in the baculovirus transfer vector, respectively, as described before (20). The β-chain contained the IAPP KS20 peptide (**Table 1**), or the mutants (KS20C2S or KS20C7S) with a C-terminal flexible linker (GGGSLVPRGSGGGGS) inserted between a signal peptide and the N terminus of the β1 domain as previously described (21, 22). We introduced a mutation at position 17 to E to enhance the binding between the peptide and IA^g7^ without adverse effects on the peptide activity based on our recent crystal structure (data not shown). The substitution increases BDC5.2.9 hybridomas (a gift from Kappler/Marrack Lab) activation more than 100 times (**Supplementary Figure S1**). The soluble IA^g7^-KS20, IA^g7^-KS20C2S, and IA^g7^-KS20C7S were expressed and purified as previously described (22). Briefly, Hi5 cells were infected with IA^g7^-KS20, IA^g7^-KS20C2S, and IA^g7^-KS20C7S recombinant baculovirus separately in spinner flasks at 19°C for 6 days. The supernatants were harvested, and the debris was removed by centrifugation. After being purified using a zipper-specific 2H11 mAb coupled with agarose resins, the purified protein was concentrated to run Superdex 200 increase to get rid of the aggregate or other contaminating proteins. For tetramer preparation, the biotinylated protein was concentrated to run Superdex 200 Increase (GE Healthcare). The purified biotinylated protein was collected to mix with streptavidin-PE for 1 h at 4°C. Then, Superdex 200 Increase was run and the purified tetramers were collected for staining. Other MHC II-peptide tetramers were gifts from Kappler/Marrack Lab. MHC I-peptide tetramer were obtained from NIH tetramer core.

### Hybridoma Generation and Screening of Monoclonal Antibody LD96.24 Against IA^g7^-KS20 Complex

Splenocytes were harvested from NOD mice with a high titer of antibody against IA^g7^-KS20 and mixed with Sp2/0 at a ratio of 5:1. After washing with balanced salt solution (BSS) to remove Fetal bovine serum (FBS), 1 mL of 40% (wt/vol) PEG 8000 was added to the cell pellet dropwise over a period of 30 s. After an additional 30 s, 1 mL of SMEM (GIBCO 11380-037) was added with mixing over a total of 1 min. Finally, 2, 3, 4, 5, and another 5 mL of Spinner Minimum Essential Medium (SMEM) media were added dropwise by mixing to the cell pellet over 1 min. The cells were then incubated at 37°C for 10 min and washed with a BSS and then resuspended in Minimum Essential Medium (SMEM) containing 10% FBS, and 100-μL aliquots distributed in flat-bottom 96-well plates. The next day, 100 μL of 2× HAT selection medium were added to each well. On days 5, 10, and 15, the plate was dumped and the new medium was changed. The testing of B hybridomas began on day 10. Supernatants from the hybridomas were screened by ELISA for antibodies specifically binding to IA^g7^-KS20 but not other MHC peptides. The positive B-cell hybridomas were selected for antibody purification. For APCs, we used the M12C3 B cell expressing IA^g7^ (M12C3^G7^) (20).

### Antibody Purification and Removal of Endotoxin in Monoclonal Antibodies

The LD96.24-secreting cells were cultured in a serum-free medium (GIBCO12045-076) and purified with protein G Sepharose beads. The endotoxin was removed with triton X-114 as previously described (23). The residual endotoxin content of the antibody was determined using Pierce™ Chromogenic Endotoxin Quant Kit (Thermo Fisher Scientific, Rockford, Illinois, US. A39552) and the endotoxin content of LD96.24 was 1.73EU/mg and A111.3 was 1.04EU/mg after removal, which was neglectable.

### Binding Affinity Assay

Fab fragments of LD96.24 were generated by papain cleavage and purified by Protein A beads. Biotinylated IA^g7^–KS20 complexes were captured in the BIAcore Streptavidin BIA sensor chip, and Fab fragments of LD96.24 were injected at various concentrations for binding affinities determined by surface plasmon resonance (Biacore2000; GE Healthcare Bio-sciences ABUppsala, Sweden) according to the manufacturer’s instructions. The overall affinity of LD96.24 was calculated with the software supplied with the instrument.

### ELISA for Binding Assay

Binding assays were conducted as previously described (17). Briefly, plates were coated with 100 μg/mL avidin overnight at 4°C. After blocking with 30% Fetal Calf Serum (FCS) and washing 3 times with PBS-T (0.1% Tween in Phosphate Buffered Saline (PBS)), adding 5 μg/mL biotinylated protein IA^g7^-KS20, IA^g7^-KS20 or IA^g7^-KS20C2S, or IA^g7^-KS20C7S and incubating for 1 h at 37°C. After washing with Phosphate Buffered Saline (PBS), adding 100 μL of different concentrations of mAb LD96.24 and incubating for 1 h at Room Temperature (RT) and then adding IgG-Alkaline Phosphatase (IgG-AP) (1:30,000) and incubating for 1 h at RT. After washing with PBST 5 times, the p-nitrophenyl phosphate (PNPP) substrate solution was added and incubated for 30–60 min and then, the OD405 was measured.

### Suppression of LD96.24 on KS20-Reactive T Cells *In Vitro*

M12C3^G7^ B-cell lines (1 × 10^5^cells/well) were used as APCs and cultured with different concentrations of peptides (**Table 1**) for 1 h at 37°C. Then different doses of LD96.24 were added, and the final concentration was 0.01, 0.1, and 0.5 mg/mL. Approximately 2 h later, different T-cell hybridomas (1 × 10^5^cells/well) were added. Then, 20–24 h later, culture supernatants were harvested and secreted IL-2 was measured by the IL-2 assay as previously described (24).

**Table 1:** The peptides used in studies.

Protein	Peptide	Amino acid Sequences
Chromogranin A	RLGL-WE14	SRLGLWSRMDQLAKELTAE
Insulin (B:9-23)	P8G	HLVERLYLVCGGEG
Insulin (B:9-23)	P8E	HLVERLYLVCGEEG
Insulin (B:9-23)	P8L	VEALYLVAGLELG
JAPP (KS20)	Wild type	KCNTATCATQRLANFLVRSS
IAPP (KS20)	V17E	KCNTATCATQRLANFLERSS
IAPP	LD11	LQTLALNAARD

### Antibody Treatment of NOD Mice

#### Early Intervention

Female NOD mice (4 weeks old) were randomly assigned to 3 groups: PBS group (n=18), isotype control IgG2a A111.3 group (0.5 mg per injection; n=18), and LD96.24 (0.5 mg per injection; n=18). Antibodies dissolved in endotoxin-free PBS (Sigma, TMS-012-A), or PBS alone, were given weekly to each mouse by intraperitoneal injection (i.p.) from 4 to 26 weeks of age. Blood glucose levels were monitored weekly from 10 weeks of age with a ReliOn Ultima blood glucose monitor (Abbott Diabetes Care, Alameda, CA), and animals were considered diabetic when the blood glucose concentration was over 250 mg/dL in two consecutive days. Serum samples were collected every 2 weeks to detect the LD96.24 level. Approximately 26 weeks later, the antibody treatment was terminated but blood glucose levels were still monitored until week 30.

#### Late Intervention

The blood glucose levels of a group of 25 female NOD mice were tested three times a week starting at 12 weeks of age. Once the concentration reached 170 mg/dL, blood glucose was repeated the next day, and those with levels higher than 170 mg/dL in two consecutive tests were randomly assigned to one of two groups that were treated with 0.5 mg of either LD96.24 (n=8) or the mouse IgG2a isotype control (n=7), respectively. Treatment was initiated immediately and continued weekly as described above. The diagnosis of diabetes is based on the blood glucose concentration over 300 mg/dL, and non-diabetic animals were treated with antibodies until they reached 23 weeks of age.

### Preparation of Pancreatic Cells of NOD Mice

Pancreatic cells were prepared following the protocol as described previously (6). Briefly, freshly isolated NOD pancreases were cut into small pieces and digested in 25 mL of balanced salt solution (BSS) containing 5% (vol/vol) FBS plus 5 μM CaCl_2_ and 100 μg/mL collagenase (C9407; Sigma) at 37° C for 15 min. The digested mixture was then washed with BSS, crushed, and passed through a 100-μm nylon mesh screen to remove residual tissue, and the cells were resuspended in the culture medium, SMEM containing 5% FBS.

### Flow Cytometry

#### MHCII-Peptide Tetramer Staining

Approximately 20 μg/mL tetramer and 1 μg/mL HAM57-597 (anti-T cell receptor(TCR)-beta chain) were added at 25 μL/well to 96-well U plates. Then, the plates were incubated at 37°C in a 10% CO_2_ incubator for 2 h and shaken to mix cells every 30 min.

#### MHC I-Peptide Tetramer Staining

For MHC I -Peptide tetramer staining, approximately 20 μg/mL tetramer was added to 25 μL each well of 96-well U plates. The plates were incubated on ice for 1 h and shaken to mix cells every 30 min.

#### Surface and Intracellular staining

After tetramers staining, the cells were stained with surface fluorescentrly labeled mAbs as follows: FITC-B220, eFluor 450-F4/80, APC eFluor 780-CD8, Alexa Fluor 700-CD4^+^, and PerCP-Cy5.5-CD44 (eBioScience). After MHC II- peptide tetramer staining and cell surface staining, cells were fixed with 4% paraformaldehyde (Thermo Scientific Cat: 00-5523, CA, US.). Intracellular staining with Foxp3-APC was done using the eBioscience Foxp3/Transcription Factor Staining Buffer Set (Thermo Fisher Scientific, Cat: 00-5523, CA, US.) according to the manufacturer’s instructions. Stained single-cell suspensions were analyzed using a Fortessa flow cytometer running FACSDiva (BD Biosciences, US). Flow Cytometry Data File Standard Version 3.0 (FSC 3.0) files were analyzed with Flowjo 10.0 Software.

### Statistical Analyses

Comparisons between two groups were performed with a two-tailed student’s t-test, and more than two groups were compared by ANOVA. Each experiment was repeated at least once to assess reproducibility. *P < 0.05, ** *P*< 0.001, *** *P*< 0.0001.

## Results

### LD96.24 Specifically Binds to the IA^g7^-KS20 Complex

**Figure 1:**
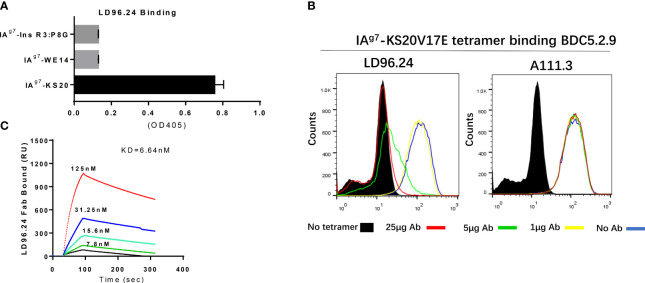
LD96.24 specifically binds on the IA^g7^-KS20V17E complex. **(A)** ELISA plates were coated with various IA^g7^–peptide complexes, including IA^g7^–KS20V17E complexes and control complexes IA^g7^-Ins R3:P8G and IA^g7^-RLGL-WE14. The binding of LD96.24 to immobilized complexes was measured with ELISA. **(B)** T-cell hybridoma BDC5.2.9, which is specific for IA^g7^-KS20, is stained with IA^g7^-KS20V17E tetramers in the presence or absence of LD96.24 or isotype antibody A111.3 and analyzed by flow cytometry. No tetramer or tetramer plus 0, 1, 5, or 25 μg LD96.24 or A111.3. **(C)** The binding kinetics of various concentrations of Fab fragments of LD96.24 to immobilized IA^g7^-KS20V17E were recorded by using surface plasmon resonance, and IA^g7^-RLGL-WE14 weas used as control.

After immunization with purified IA^g7^-KS20 proteins and adjuvants 3 times in 5 NOD mice, antibodies against IA^g7^-KS20 complexes could be detected in all mice. We chose one mouse with the highest antibody titer for hybridoma generation. The supernatants of B-cell hybridomas were used for screening. We coated IA^g7^-KS20 and IA^g7^-RLGL-WE14 to screen the IA^g7^-KS20 specific antibodies. The antibody produced by clone 96 named LD96.24 specifically bound IA^g7^-KS20 complex, but not IA^g7^- RLGL-WE14 complex. We used this mAb in all subsequent experiments. The isotype analysis showed that the isotype of LD96.24 is IgG2a, which might mediate antibody-dependent cell cytotoxicity (ADCC) *in vivo*. First, the specificity of LD96.24 on IA^g7^-KS20 was analyzed (the peptide sequences in these studies are listed in **Table 1**). As shown in **Figure 1A**, LD96.24 specifically bound IA^g7^-KS20 complex, but not IA^g7^-RLGL-WE14 or IA^g7^-Ins B:9-23 P8G complex. Next, we used an mAb against endotoxin A111.3 (a gift from Kappler/Marrack lab) as an IgG2a isotype control to test if LD96.24 can block the staining of IA^g7^-KS20 tetramers to BDC5.2.9 T cells. As we expected, LD96.24, but not A111.3, had the ability to block IA^g7^-KS20 tetramers to stain the BDC5.2.9 T-cell hybridomas. Approximately 25 μg LD96.24 almost completely inhibited the binding of IA^g7^-KS20 tetramer on BDC5.2.9 (**Figure 1B**). Finally, the surface plasmon resonance experiments showed that LD96.24 specifically binds IA^g7^-KS20, with a high binding affinity (Kd = 6.64 nM) (**Figure 1C**).

### LD96.24 Inhibits the KS20-Specific T-Cell Responses

**Figure 2:**
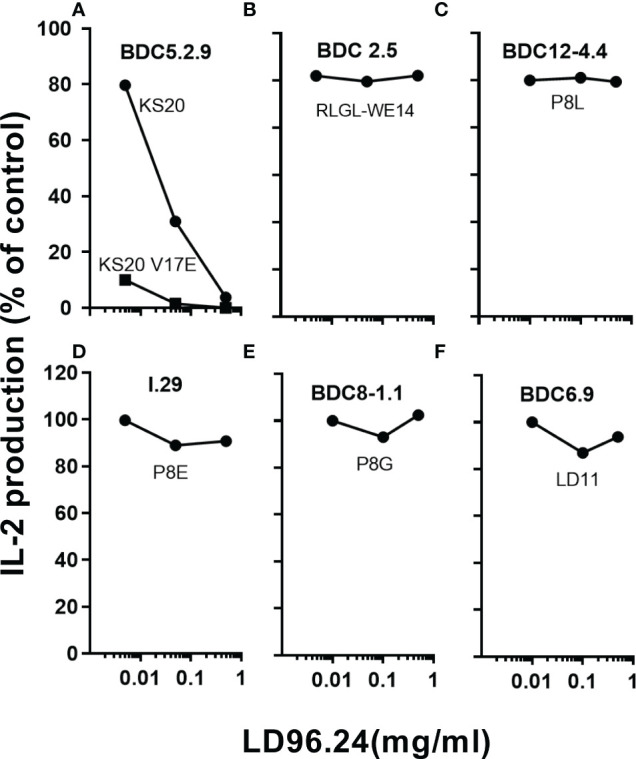
LD96.24 specifically inhibits the KS20-specific T-cell responses. The inhibition of LD96.24 on different autoantigen-reactive T cells were assessed *in vitro*. As shown in the methods, different T cells including BDC5.2.9 specific for KS20 **(A)**, BDC2.5 specific for ChgA (RLGL-WE14 peptide) **(B)**, BDC12-4.4 specific for Ins P8L **(C)**, I.29 specific for Ins P8E **(D)**, BDC8-1.1 specific for Ins P8G **(E)**, and BDC6.9 specific for LD11 **(F)** were incubated with APCs loaded with different peptides and different concentrations of LD96.24 for 24 h at 37°C, and IL-2 production was tested with the HT-2 assay. The results are shown as the percentage of the response remaining compared with the no LD96.24 control.

Since LD96.24 can specifically block the IA^g7^-KS20 tetramer staining of BDC5.2.9 T cells, we tested if LD96.24 could specifically inhibit the activation of KS20-reactive T-cell hybridomas, BDC5.2.9. The results showed that LD96.24 inhibited the IL-2 secretion by specifically inhibiting T-cell responses to KS20 peptides in a dose-dependent manner (**Figure 2A**) but had no effects on ChgA-responsive BDC2.5 T-cell hybridomas (**Figure 2B**), Ins P8L–reactive BDC12-4.4 T-cell (**Figure 2C**), type A (**Figure 2D**) and type B (**Figure 2E**) ins B:9-23–reactive T-cell, and LD11-reactive T-cell hybridomas, BDC6.9 (**Figure 2F**). LD96.24 can also inhibit the other two KS20-reactive T-cell lines, BDC-5/S3.4 and BDC-9/S3.5 (data not shown). These results indicated that LD96.24 specifically inhibited the KS20-specific T-cell *in vitro*.

### KS20 Disulfide Bridges Are Required for the Binding of LD96.24

**Figure 3:**
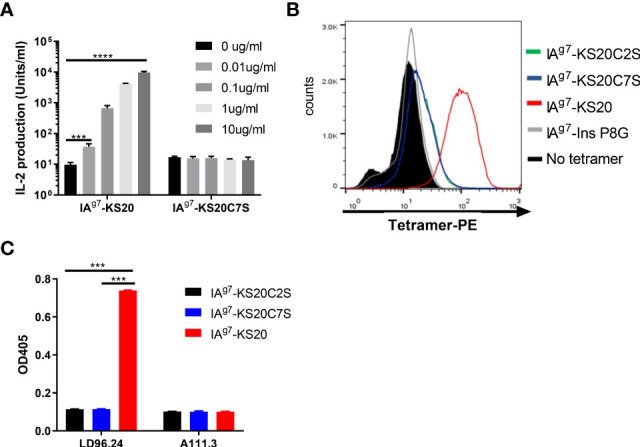
LD96.24 specifically binds the IA^g7^-KS20 disulfide loop at the N terminal. **(A)** M12C3^G7^ bears KS20 or KS20C7S peptides to stimulate BDC5.2.9 at different concentrations: 0, 0.01, 0.1, 1, and 10 μg/mL. Approximately 24 h later, supernatants were collected for the HT-2 assay. **(B)** IA^g7^-KS20C2S/C7S tetramers were used to stain BDC5.2.9. IA^g7^-KS20 as a positive control and IA^g7^-Ins P8G as a negative control. **(C)** Binding between mAb LD96.24 and IA^g7^-KS20/KS20C2S/KS20C7S molecules were measured by ELISA as described in Materials and Methods. A111.3 antibody was used as a control. Results are means ± SEM of triplicate wells. Statistical analysis was performed using one-way ANOVA with Tukey’s multiple comparison test. *P < 0.05, ** P < 0.001, *** P < 0.0001.

The previous IAPP peptide truncation results suggested that peptides NS18 (NTATCATQRLANFLVRSS) and TS17 (TATCATQRLANFLVRSS) could not activate BDC5.2.9 compared to KS20 (KCNTATCATQRLANFLVRSS) and peptide CS19 (CNTATCATQRLANFLVRSS) (10). Deleting cysteine 2 in peptide NS18 and TS17 completely abolished the activation of BDC5.2.9 (10). MHC molecules normally bind to short linear fragments of proteins to be examined by αβ TCRs. This leads to a relatively flat TCR VαVβ recognition surface. TCRαβ heterodimers normally dock onto the MHC/peptide complex using a common diagonal mode such that the Vα domain contacts the MHC α2 helix and the Vβ domain overlays the α1 helix (25, 26). The diagonal model of TCR/MHC/peptide complex recognition was needed for CD4^+^ and CD8 binding, which may be important for a productive TCR–CD3 complex formation (27). As disulfide bonds are formed, the shape and charge of the peptide antigens would change drastically. This leads to the unusual binding modes of T cells and elicits T-cell responses. To confirm that the disulfide loop is the primary binding site to the TCR of BDC5.2.9, we mutated the Cys2 or Cys7 of KS20 to Ser to break the disulfide bridge and use the B cell M12C3^G7^ as APCs to stimulate BDC5.2.9 in the presence of KS20 peptide or KS20C7S peptide (20). The results showed that non-disulfide KS20C7S peptide could not activate BDC5.2.9, even at the concentration of 10 μg/mL (**Figure 3A**). This indicated that the KS20 disulfide loop is crucial for BDC5.2.9 activation. We also generated the IA^g7^-KS20C2S/C7S tetramers to evaluate the interactions between IA^g7^-KS20C2S/C7S and BDC5.2.9. The results showed that IA^g7^-KS20C2S/C7S tetramers stained the BDC5.2.9 T-cell weakly (**Figure 3B**) and confirmed that the disulfide loop is the major binding site to TCR of BDC5.2.9. Due to the prominence of the disulfide bridge on the face of MHC, we believed that LD96.24 might bind this disulfide loop and block the interactions between IA^g7^-KS20 and the TCR of BDC5.2.9. We used the ELISA assays to test the binding between LD96.24 and IA^g7^-KS20 and its variant proteins. The results showed that after mutating the Cys2 or Cys7 of KS20 to Ser to break the disulfide bridge, LD96.24 has no binding affinity with IA^g7^-KS20 Cys variants (**Figure 3C**).

### LD96.24 Delays the Onset of T1D

**Figure 4:**
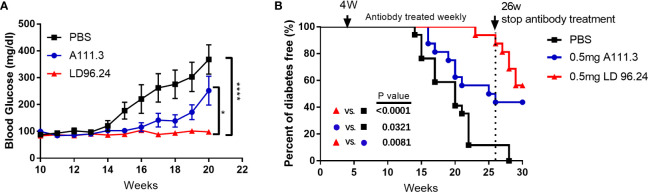
Monoclonal antibody LD96.24 delays the onset of T1D. Groups of 4-week-old female NOD mice were treated weekly with PBS n = 18; black squares), 0.5 mg of LD96.24 (n = 18; red triangles), and 0.5 mg of isotype control antibody A111.3 (n = 18; blue circles) until 30 weeks. The blood glucose levels of each group were monitored weekly from 10 weeks of age. Mice were considered diabetic after two consecutive blood glucose concentrations ≥250 mg/dL. **(A)** The changes in the blood glucose level in each group. Data are means ± SEM. **(B)** The percentages of the remaining diabetes-free mice of each group are shown. The log-rank (Mantel–Cox) test was used for the survival plots. P-values were determined by using the χ2 log-rank test.

LD96.24 would be an ideal tool to test how disulfide-modified antigens contribute to the development of T1D. Because of the high specificity and affinity between LD96.24 and the IA^g7^-KS20 complex and the inhibition on the KS20-reactive T-cell activation *in vitro*, we decided to test if LD96.24 would modulate the autoimmune responses by inhibiting KS20- reactive T cells *in vivo.* We treated the female NOD mice with LD96.24 or isotype control antibody A111.3 weekly starting at 4 weeks of age. The average half-life of serum IgG2a was 6–8 days, as previously determined. The level of LD96.24 in the serum was maintained over 150 μg/mL from 12 to 20 weeks, while it decreased to below 150 μg/mL after 20 weeks (**Supplementary Figure S2**). The blood glucose level of the LD96.24 antibody–treated group was not significantly changed from 10 to 20 weeks, while the PBS group and isotype control antibody group increased significantly (**Figure 4A**). Compared with the PBS and isotype control groups, LD96.24 significantly delayed the onset of T1D at the dose of 0.5 mg/mouse till 26 weeks (**Figure 4B**). Strikingly, there were 100% diabetes-free NOD mice at week 23 and 88.9% at week 26, while the isotype control antibody group started to develop diabetes at week 16 and had 50% diabetes-free NOD mice at week 26 (**Supplementary Figures S3A–C**). To confirm that the delay of diabetes was due to LD96.24 administration, we stopped the antibody treatment after 26 weeks and monitored the blood glucose until 30 weeks. The results showed that the diabetes-free NOD mice decreased from 87.5% to 56% in just 4 weeks. However, in the isotype control group, the diabetes-free NOD mice were maintained at the level of 43% until 30 weeks. *In vivo* experiments showed that LD96.24 effectively delayed diabetes onset at the early treatment stages.

### LD96.24 Suppresses T-Cell and B-Cell Infiltration Into the Pancreas

**Figure 5:**
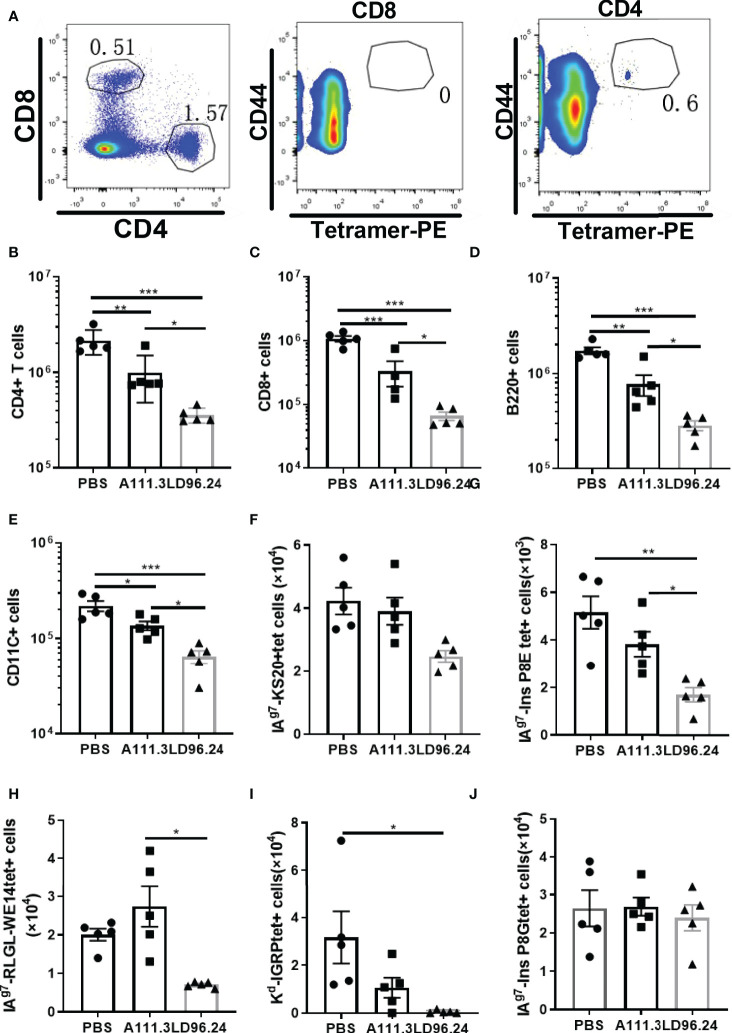
The impact of LD96.24 on lymphocyte changes in the pancreas. Three groups (5 NOD mice/group) were treated weekly from age 4 weeks with PBS, 0.5 mg A111.3 or LD96.24, and pancreatic cells were isolated at age 10 weeks. **(A)** Representative scatter diagram of the gating strategy for tetramer-positive CD4+ T cells. First gating on live, B220-, F4/80-, CD8-, CD44high, and CD4+ T cells, then using CD8+ T cells to set gate to examine the tetramer-positive CD4^+^T cells. **(B-D)** The average number of CD4^+^T cells **(B)**, CD8 T cells **(C)**, B cells **(D)**, and DCs **(E)** in the pancreas. **(F-J)** Infiltrated tetramer-positive CD4^+^T cells and CD8 T cells, including IAPP KS20-reactive T cells **(F)**, InsP8E-reactive T cells **(G)**, RLGL-WE14-reactive T cells **(H)**, Kd-IGRP-reactive T cells **(I)**, and InsP8G-reactive T cells **(J)** in the pancreas were detected with flow cytometry. Data are the means ± SEM of each group. Statistical analysis was performed using one-way ANOVA with Tukey’s multiple comparison test. *P < 0.05, ** P < 0.001, *** P < 0.0001.

Islet lymphocyte infiltration is the hallmark of T1D. Many cell types are involved in the pathogenesis of diabetes, including T cells, B cells, dendritic cells (DCs), and macrophages. Islet antigen–specific CD4^+^ and CD8^+^ T cells are the principal drivers of disease initiation and progression. The *in vitro* experiment showed that LD96.24 inhibited the interaction between APCs and pathogenic T-cell hybridoma BDC5.2.9. The *in vivo* experiment also confirmed that LD96.24 significantly delayed the onset of T1D in NOD mice when treated from week 4. One possibility would be that LD96.24 might be cytotoxic for APCs (B cells, macrophages, and DCs) *in vivo* because the subclass of LD96.24 is IgG2a, which may mediate complement fixation and ADCC. The results ruled out that there were no changes in the level of surface IA^g7^ expression in B cells (**Supplementary Figure S4A**) and there were no significant differences in the proportion of B220^-^, CD11c^-^, and CD11b^-^positive cells in the spleen of LD96.24-treated mice (**Supplementary Figures S4B–D**). The recruitment of circulating APCs into the pancreas may further enhance the presentation of the IAPP and induce the KS20-reactive T cells in the pancreas. Previous data showed that islet-specific T cells in NOD mice are regulated by IA^g7^ expressing B-cell-mediated Ag presentation (28). We predicted that LD96.24 might delete APCs bearing KS20, which leads to the decreased number of KS20-reactive T cells in the pancreas. We determined the numbers of APCs in the pancreas after LD96.24 treatment. As **Figures 5A-J** shows, the numbers of CD4^+^ (**Figure 5B**) and CD8 T cells (**Figure 5C**), B cells (**Figure 5D**), and DCs (**Figure 5E**) significantly decreased in the pancreas of the LD96.24 treatment group compared with the PBS group and A111.3 control group. I-A^g7^ tetramers loaded with different peptides, including KS20, RLGL-WE14, Ins B:9-23 P8G, and Ins B:9-23 P8E, were used to detect the infiltrated T cells. The results showed that the numbers of IAPP (**Figure 5F**), Ins B:9-23 P8E (**Figure 5G**), chromogranin A (**Figure 5H**), and Islet-specific glucose-6-phosphatase catalytic subunit-related protein (IGRP) (**Figure 5I**) specific T cells decreased in the pancreas after LD96.24 treatment compared with the PBS group and A111.3 group. There were no significant cell number changes of Ins B:9-23 P8G–reactive T cells in the pancreas (**Figure 5J**).

### LD96.24 Changes the Balance Between Regulatory T Cells and Effector T Cells

**Figure 6:**
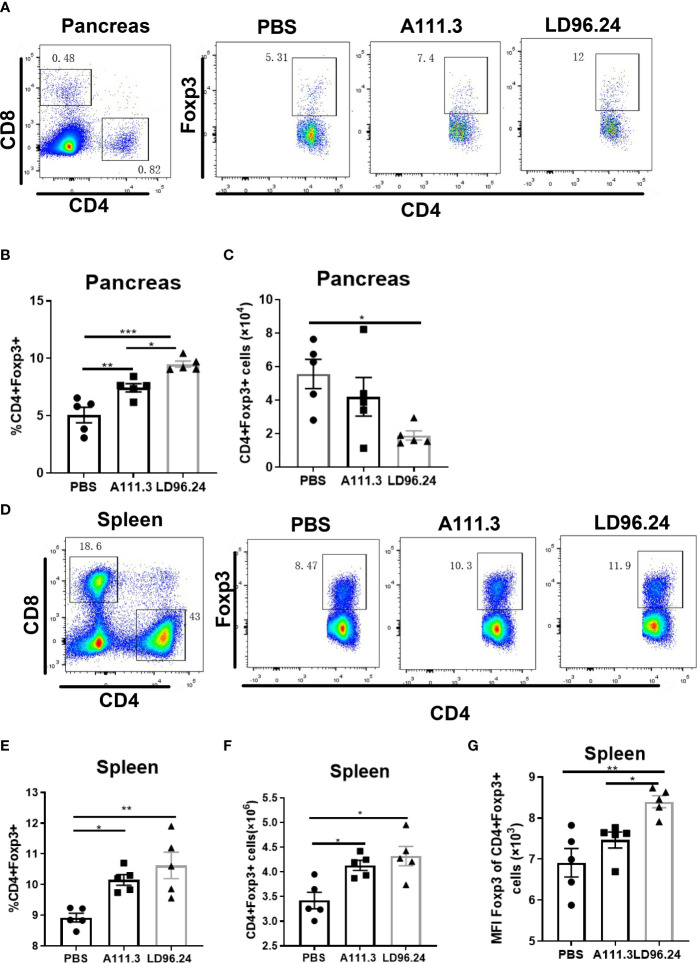
The impact of LD96.24 on CD4^+^Treg in pancreas and spleen. Three groups (5 NOD mice/group) were treated weekly from age 4 weeks with PBS, 0.5 mg A111.3 or LD96.24, and pancreatic cells were isolated at age 10 weeks. Cell surface antibodies were stained with BV605-B220, APC eFluor 780-CD8, Alexa Fluor 700-CD4^+^, and PerCP-Cy5.5-CD44. Then, intracellular staining with APC-Foxp3 was done using the eBioscience Foxp3/Transcription Factor Staining Buffer Set (Thermo Fisher Scientific) according to the manufacturer’s instructions. Stained single-cell suspensions were analyzed using a Fortessa flow cytometer running FACSDiva (BD Biosciences). **(A-C)** The percentages and cell number of CD4^+^Foxp3+Tregs in the pancreas. **(D-F)** The percentages and cell number of CD4+Foxp3+Tregs in the spleen. **(G)** The mean fluorescence intensity (MFI) of CD4+Foxp3+Tregs in the spleen. Data are the means ± SEM of each group. Statistical analysis was performed using one-way ANOVA with Tukey’s multiple comparison test. *P < 0.05, ** P < 0.001, *** P < 0.0001.

**Figure 7:**
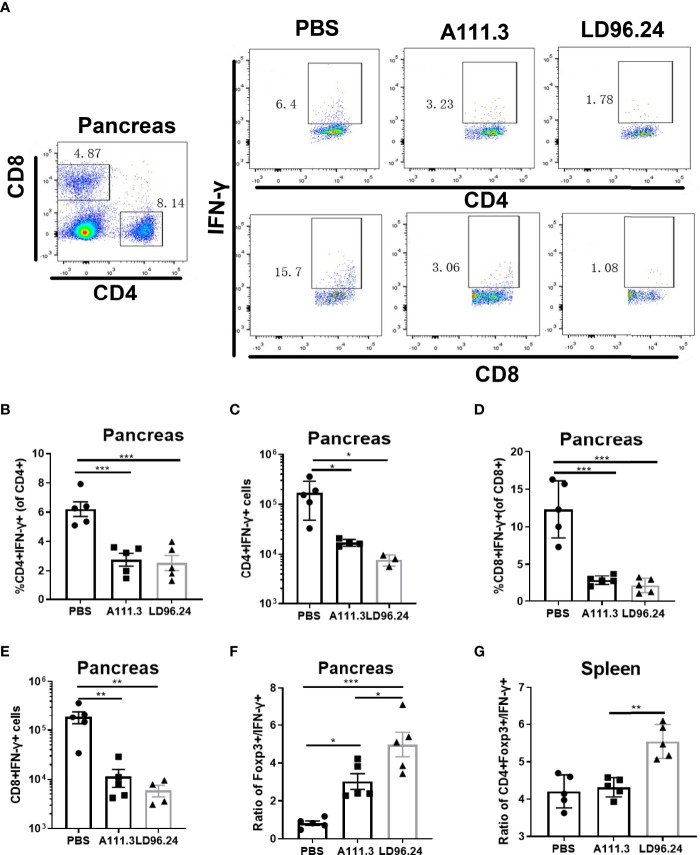
LD96.24-treated mice have an increased CD4+Foxp3+ to CD4^+^IFN-γ+ T- cell ratio in the pancreas and spleen. Three groups (5 NOD mice/group) were treated weekly from age 4 weeks with PBS, 0.5 mg A111.3 or LD96.24, and pancreatic cells were isolated at age 10 weeks. After stimulation with cell-activation cocktail (cat#: 423301) for 4 h. Cell surface antibodies were stained with BV605-B220, APC eFluor 780-CD8, Alexa Fluor 700-CD4^+^, and PerCP-Cy5.5-CD44. Then, intracellular staining with FITC-IFN-γ was done using the eBioscience Foxp3/Transcription Factor Staining Buffer Set (Thermo Fisher Scientific) according to the manufacturer’s instructions. Stained single-cell suspensions were analyzed using a Fortessa flow cytometer running FACSDiva (BD Biosciences). **(A)** Representative scatter diagram of CD4^+^IFN-γ+ and CD8+IFN-γ+ in different groups. **(B, C)** The percentages and cell numbers of CD4^+^IFN-γ+ T-cells in the pancreas. **(D, E)** The percentages and cell numbers of CD8+IFN-γ+ T-cells in the pancreas. **(F, G)** The ratio of CD4+Foxp3+/CD4^+^IFN-γ+ in the pancreas **(F)** and spleen **(G)**. Data are the means ± SEM of each group. Statistical analysis was performed using one-way ANOVA with Tukey’s multiple comparison test. *P < 0.05, ** P < 0.001, *** P < 0.0001.

CD4^+^ regulatory T-cells (Treg) cells expressing the transcription factor Foxp3 play a critical role in preventing systemic autoimmunity. The autoimmune destruction of the pancreatic islets in T1D is mediated by increasing Teffs and decreasing Treg T lymphocytes. This would result in a significant decrease in the Foxp3^+^ Tregs/IFN-γ^+^ Teffs ratio as the anti-CD3 antibody can induce the inactivation of conventional T cells and the expansion of Treg populations (29). We tested if LD96.24 inhibits the development of insulitis by suppressing autoantigen-specific T cell infiltration and changing the ratio of Foxp3^+^ Tregs/IFN-γ^+^ Teffs. The results showed that the LD96.24-treated group significantly increased the percentages of Foxp3^+^ Treg in the pancreas (**Figures 6A, B**). However, the cell numbers of CD4^+^Treg significantly decreased in the pancreas compared with the PBS group because of the decreased total CD4^+^ T cells in the pancreas (**Figure 6C**). Both the percentages and cell numbers of Treg significantly increased compared with the PBS group in the spleen (**Figures 6D–F**). LD96.24 also significantly elevated the level of Foxp3^+^ in Treg in the spleen (**Figure 6G**). The previous data showed that the levels of Foxp3 in Treg may be associated with the inhibitory function on effector T-cell activation (30, 31). Our results indicated that LD96.24 not only increased the proportion of Treg but also enhanced the inhibitory function of Treg in spleen. Next, we tested the impact of LD96.24 on effector T-cells (Teffs) in the pancreas. The results showed that A111.3 and LD96.24 can both significantly decrease the percentages and cell numbers of CD4^+^IFN-γ^+^ and CD8^+^IFN-γ- in the pancreas (**Figures 7A–E**), which may account for A111.3 and could also delay autoimmune diabetes compared with the PBS group. LD96.24 can significantly decrease the cell numbers of CD4^+^IFN-γ- Teff in the spleen but did not significantly decrease the cell numbers of CD8^+^IFN-γ- in the spleen (**Supplementary Figures S5A–E**). We also calculated the ratio of CD4^+^Foxp3^+^/CD4^+^IFN^+^γ-, and the results showed that LD96.24 significantly increased the Foxp3^+^ Tregs/IFN^-^γ^+^ Teffs ratio in the pancreas (**Figure 7F**) and spleen (**Figure 7G**) compared with the isotype control antibody A111.3 and PBS group. These results indicated that LD96.24 changed the balance between Treg and Teff, which may play an important role in delaying the development of T1D.

### LD96.24 Delays the Progression of Type 1 Diabetes at a Late Prediabetic Stage in NOD Mice

**Figure 8:**
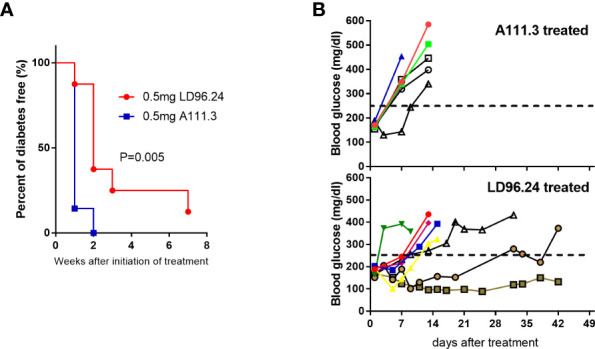
Treatment of NOD mice with LD96.24 delays progression to T1D. Groups of 12-week-old female NOD mice were monitored for blood glucose until they were over 170 mg/mL. Then, they were treated weekly with 0.5 mg LD96.24 (n=8) or 0.5 mg A111.3 (n=7) until the animals reached 23 weeks of age. **(A)** Blood glucose levels of individual mice receiving IgG2a isotype control antibody A111.3 (upper) or LD96.24 (lower). **(B)** Percentages of diabetic-free mice after LD96.24 and A111.3 antibody treatment. P-values were determined by using the χ2 log-rank test.

The data above showed that LD96.24 could delay the development of diabetes when treatment is started at an early stage. We also wanted to know if LD96.24 could be effective at late-stage intervention when the blood glucose concentration is over 170 mg/mL. We started to test the blood glucose level at 8 weeks of age. Once the blood glucose level reached more than 170 mg/mL in two consecutive days, we immediately treated the mice with weekly single injections until the blood glucose concentration was over 300 mg/dL or the age of animals reached 23 weeks. As shown in **Figure 8A**, the LD96.24 therapy significantly delayed the development of diabetes (*P*=0.005). Individual weekly blood glucose levels for each NOD mouse are shown in **Figure 8B**. Seven out of eight NOD mice were diabetes-free within 1 week. One out of four NOD mice remained non-diabetic until 23 weeks. In contrast, all the mice treated with isotype control antibodies developed diabetes within 2 weeks (**Figure 8B**). The results shown above indicated that treatment with LD96.24 at a late stage could significantly delay the progression of diabetes in NOD mice.

## Discussion

Autoreactive T-cells are central to the development of T1D. Multiple T-cell-reactive islet autoantigens are present in pancreatic β cells. The Ins B:9-23 peptide is a major autoantigen for pathogenic CD4^+^ T-cells and is believed to be critical to the disease initiation prior to other antigens entering the disease (32–35). Ninety percent of Ins-specific CD4^+^ T-cell clones from the islets of NOD mice are specific to the Ins B:9-23 peptide (36). Changing a single amino acid of the B:9-23 peptide can genetically prevent autoimmune diabetes in NOD mice (37). The post-translational peptide splicing of the 9-23 peptide most likely accounted for the potency of the islet-generated peptide (5, 20, 38, 39). Ins autoantigens have been extensively studied and are the major targets of antigen-specific immunotherapies for T1D (40, 41). However, the disulfide-modified IAPP peptide could also stimulate the highly diabetogenic islet-specific CD4^+^T cell clones (9). The IAPP KS20 epitope was previously shown to be the epitope for the prototypic diabetogenic CD4^+^T cell clone BDC5.2.9, originally isolated from the infiltration of NOD mice pancreas (9). Multiple KS20-specific T-cell clones have been demonstrated to be capable of the adoptive transfer of diabetes (10). The KS20-specific CD4^+^T-cells can be detected in the pancreas of prediabetic and diabetic NOD mice. We found that KS20-specific T-cells (Teffs and Tregs) in the pancreas increased with the development of T1D in NOD mice (data not shown). In this study, we generated an mAb, LD96.24, which is highly specific to the disulfide bridge of the KS20 peptide when bound to IA^g7^ and effectively delayed the diabetes onset. Strikingly, the treatment study showed that the mAb 100% prevented T1D till week 24 at early administration. The efficacy of LD96.24 was unexpectedly greater than the mAbs against IA^g7^ bound to the Ins B:9-23 peptide (17, 18). The diabetes-free NOD mice dropped from 87.5% to 56% in 4 weeks after we stopped the antibody treatment at week 26 in the LD6.24 treatment group (**Figure 4**). This confirmed that the T1D delay was mediated by this mAb. In contrast, in the isotype control group, diabetes-free NOD mice maintained at the level of 43% until week 30 after stopping administration, which suggested that LD96.24 specifically suppressed autoantigen-reactive T-cell activation and significantly delays the onset of T1D in the late stage. The treatment of NOD mice with mAb LD96.24 significantly delayed the progression of T1D also in the late-intervention experiment (**Figure 8**). The data implied that the LD96.24 could block disease progression in NOD mice that have already become autoantibody positive. This could be important for the translational use of the antibody in humans.

The mechanisms of the high effectiveness of the T1D delay in NOD mice by mAb LD96.24 were multifaceted. LD96.24 reduced the infiltrated T-cells, B cells, and DCs in the pancreas. The reduction of B cells and DCs may be directly associated with the decreased number of KS20-specific T cells. B cells and DCs expressing IA^g7^ molecules are critical APCs for the initiation of T-cell-mediated autoimmune diabetes in NOD mice (28, 42, 43). The elimination of the APCs in the pancreas could induce T-cell anergy, and perturbate the T helper (Th) 1/Th2 balance. LD96.24 not only reduces the IAPP-specific T-cells but also chromogranin A and Ins-specific T-cells. Similar Teff effects were observed in Ins-specific mAb287 treated NOD mice (17). Like LD96.24, mAb287 inhibited the infiltration of different autoantigen-specific T-cells into the pancreas. Interestingly, the percentages of Tregs in the pancreas and spleen expanded after mAb treatment. It was the first evidence showing that the APC targeting mAb could increase Treg populations. Tregs play an important role in controlling autoimmune diseases, including T1D, by controlling autoreactive T-cells (44). Treatment with mAbs targeting CD3 induced permanent remission in NOD mice (45), which was associated with the expansion of Treg populations (29). Due to the promiscuous T-cell specificities, anti-CD3 mAbs could trigger a systemic release of multiple cytokines causing acute toxicity (46, 47). Compared with anti-CD3 mAb, LD96.24 only targets self-antigen-specific T-cells in the NOD mice treatment and modulates T1D disease without the pleiotropic effects of non-selectivity. LD96.24 engagement may induce a “tolerogenic” phenotype in APCs expressing pathogenic IA^g7^-KS20 complexes by secreting tolerogenic cytokines, giving rise to Treg cells. However, we could not completely rule out the involvement of the thymic differentiation of Tregs. We speculate that LD96.24 may not only induce tolerogenic immune response against IAPP but also induce tolerance to other autoantigens through a type of linked suppression (48, 49). The somewhat increase of the Tregs with the isotype control antibody compared with the PBS group was perhaps a consequence of the non-specific Fc receptor binding of LD96.24 (50). LD96.24 significantly decreased the proportion and numbers of CD4^+^IFN-γ^+^ and CD8^+^IFN-γ^+^ T-cells in the pancreas, while it increased the proportion of Tregs in the pancreas. We found that LD96.24 significantly increased the Foxp3^+^ Tregs/IFN-γ^+^ Teffs ratio in the pancreas and spleen. The ratio of Foxp3^+^ Tregs/IFN-γ^+^ Teffs may contribute to the delay of diabetes. Changing the balance between Teffs and Tregs in the pancreas may reduce the destruction of autoreactive T cells. Similarly, the nanoparticles containing an Ins-ChgA hybrid peptide could significantly increase the ratio of Ag-specific Foxp3^+^Tregs to IFN-γ^+^ Teffs (48, 51, 52). Our data showed that LD96.24 increased the ratio of Foxp3^+^ Tregs to IFN-γ^+^ Teffs and delayed the development of T1D, which indicated that the ratio of Foxp3^+^ Tregs T-cells to IFN-γ^+^ Teffs might be an indicator of the development of T1D. The sustainable Teff suppression and Treg upregulation suggested that the LD96.24 treatment could have long-lasting protective effects in the presence of this mAb.

Ins-specific T-cells are important for the initiation of the disease in NOD mice. In humans, the reactivities to different non-conventional antigens may initiate the T1D (53). Increasing evidence suggests that ROS derived from NADPH oxidase and ER stress play a central role in the onset of T1D both in mice and humans (54, 55). These factors may affect the post-translational modification and neoantigen formation. The increased oxidative stress leads to dysregulation in the redox balance by elevating the disulfide level, breaking the normal thiol-disulfide equilibrium. The ROS species are primary molecules that cause oxidative damage above physiological levels. Thiol plays a vital role in preventing the formation of the oxidative stress state in cells. Thiol units can be oxidized by ROS and regenerate into reversible disulfide bonds. Here, we showed the first functional mAb targeting the disulfide loop of the KS20 peptide and delayed the development of T1D. The results showed that the disulfide-modified IAPP could play a significant role in T1D development. This work not only deepened our understanding of the mechanism of disulfide-modified IAPP-reactive T-cell activation but also offered a novel avenue for the immune intervention of T1D. LD96.24 could tilt the thiol/disulfide balance by “neutralizing” the disulfide bonds. A global profiling approach for thiol oxidation to identify perturbed Cys containing protein redox states in the pancreas at different stages of T1D would be very useful (56). LD96.24 was able to suppress the pancreas infiltration of T-cells recognizing multiple epitopes from β-cell proteins including Ins autoantigens. This implied that IAPP-like disulfide-modified autoantigens were present and played an important role in the T1D pathogenesis probably through epitope spreading. IAPP-/- NOD mice still developed T1D (10). Without IAPP, the other potential disulfide-modified autoantigens may still utilize other pathways to spread the epitopes. Our study showed that targeting the disulfide-containing autoantigens could robustly inhibit disease development.

The disulfide-modified autoantigens as important diabetogenic antigens have been largely unnoticed. The calcitonin-gene related peptide (CGRP) family is a group of disulfide-containing peptide hormones consisting of IAPP, calcitonin, adrenomedullin, and CGRP. All CGRP family peptides share a common motif CXXXXC, near the N-termini both in mice and humans. Due to the similarity, LD96.24 mAb could be cross-reactive to other disulfide-containing CGRP family peptides. The pathogenic T-cells reactive to other CGRP family peptides need to be explored. This could explain why the intervention targeting the disulfide autoantigens was highly effective in the T1D treatment. The amino acid sequences of human and mouse KS20 peptides only differ by one amino acid. The HLA-DQ8 showed striking structural similarities with the NOD mouse IA^g7^ class II molecule and has a comparable peptide-binding preference (57, 58). We speculate that the KS20 specific T cells may exist in T1D patients with HLA-DQ8 and contribute to the development of T1D. Given the similarity of IAPP between humans and mice, we believed disulfide-containing antigens could be important T-cell epitopes in human T1D. More studies are required to unravel the tolerogenic mechanisms of LD96.24 mAb for developing effective immunotherapies.

## Data Availability Statement

The original contributions presented in the study are included in the article/**Supplementary Material**. Further inquiries can be directed to the corresponding author.

## Ethics Statement

The animal study was reviewed and approved by National Jewish Health.

## Author Contributions

Conceptualization, WL, YZ, and SD; methodology, WL, YZ, RL, YW, LC, and SD performed the experiments; WL and SD wrote and edited the manuscript. All authors have read and agreed to the published version of the manuscript.

## Funding

This work was supported by NIH Grants 5T32-AI-074491 (to YW), R56 AI15348 (to SD), R21 AI149655 (to SD) and DRC P30 grant, P30 DK116073 and a grant from The ALSAM Foundation.

## Conflict of Interest

The authors declare that the research was conducted in the absence of any commercial or financial relationships that could be construed as a potential conflict of interest.

## Publisher’s Note

All claims expressed in this article are solely those of the authors and do not necessarily represent those of their affiliated organizations, or those of the publisher, the editors and the reviewers. Any product that may be evaluated in this article, or claim that may be made by its manufacturer, is not guaranteed or endorsed by the publisher.

## Supplementary Material

Supplementary Figure 1 Mutation at position V17 to E of KS20 peptide enhances the activation of BDC5.2.9. M12C3^G7^ B cell lines (1×10^5^cells/well) were used as APCs and cultured with different concentrations of KS20 peptide or KS20V17E peptide. Then add BDC5.2.9 (1×10^5^/well). 20-24 h later, culture supernatants were then harvested, and secreted IL-2 was measured by IL-2 assay.

Supplementary Figure 2 Serum levels of LD96.24 in different groups. Serum samples in different groups were collected every 2 weeks with 3-d delay after antibody treatment. LD96.24 was measured by using IA^g7^-KS20 coated plates as described in Materials and methods.

Supplementary Figure 3 Related to **Figure 4**. Blood glucose levels of individual mice in each group. Blood glucose of each mouse was measured weekly in PBS group **(A)**, isotype control A111.3 group **(B)** or LD96.24 group **(C)**.

Supplementary Figure 4 Related to **Figure 5**. Treatment with LD96.24 has no effect on distributions of T cells and APCs in the spleen. 4w female NOD mice (n=5) were treated with PBS, 0.5 mg LD96.24 or control antibody A111.3 weekly and sacrificed at 10 weeks of age. Splenocytes were prepared and stained with antibodies of B220, CD11c, and CD11b. **(A)** The mean fluorescence intensities of IA^g7^ in B cells. **(B-D)** The percentages of B220+ **(B)**, CD11c+ **(C)** and CD11b+ **(D)** in total splenocytes are shown. Data are means ± SEM of each group.

Supplementary Figure 5 related to **Figure 7**. The impact of LD96.24 on CD4^+^IFN-γ+ and CD8+IFN-γ+ in spleen. The animal experiment is the same as **Figure 7**. described. The splenocytes were isolated for staining. **(A)** Representative scatter diagram of CD4^+^IFN-γ+ and CD8+IFN-γ+ in different groups. **(B, C)** The percentages and cell numbers of CD4^+^IFN-γ+ T cells in the spleen. **(D, E)** The percentages and cell numbers of CD8+IFN-γ+ T cells in the spleen. Data are means ± SEM of each group. Statistical analysis was performed using one-way ANOVA with Tukey’s multiple comparisons test. *P < 0.05, ** P< 0.001, *** P< 0.0001.
