# Supplementary material for: A Novel Tolerogenic Antibody Targeting Disulfide-Modified Autoantigen Effectively Prevents Type 1 Diabetes in NOD Mice
Source: Front Immunol. 2022 Aug 11;13:877022. doi: 10.3389/fimmu.2022.877022 (PMC9406144; doi:10.3389/fimmu.2022.877022)
Supplement: Supplementary Figure 1 — Mutation at position V17 to E of KS20 peptide enhances the activation of BDC5.2.9. M12C3G7 B cell lines (1×10^5^cells/well) were used as APCs and cultured with different concentrations of KS20 peptide or KS20V17E peptide. Then add BDC5.2.9 (1×10^5^/well). 20-24 h later, culture supernatants were then harvested, and secreted IL-2 was measured by IL-2 assay. [file Presentation_1.pptx]

## Slide 1
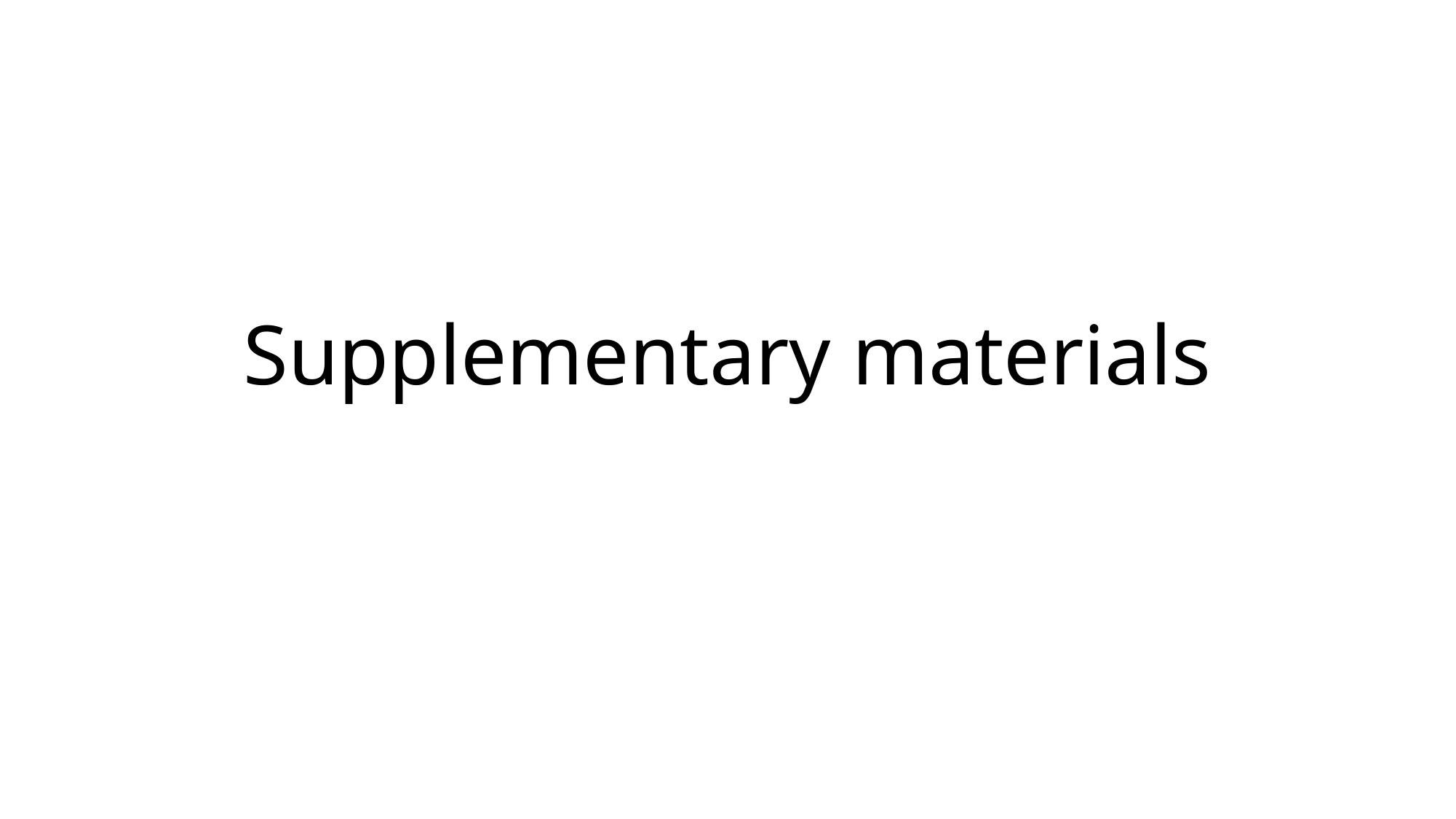

Supplementary materials

## Slide 2
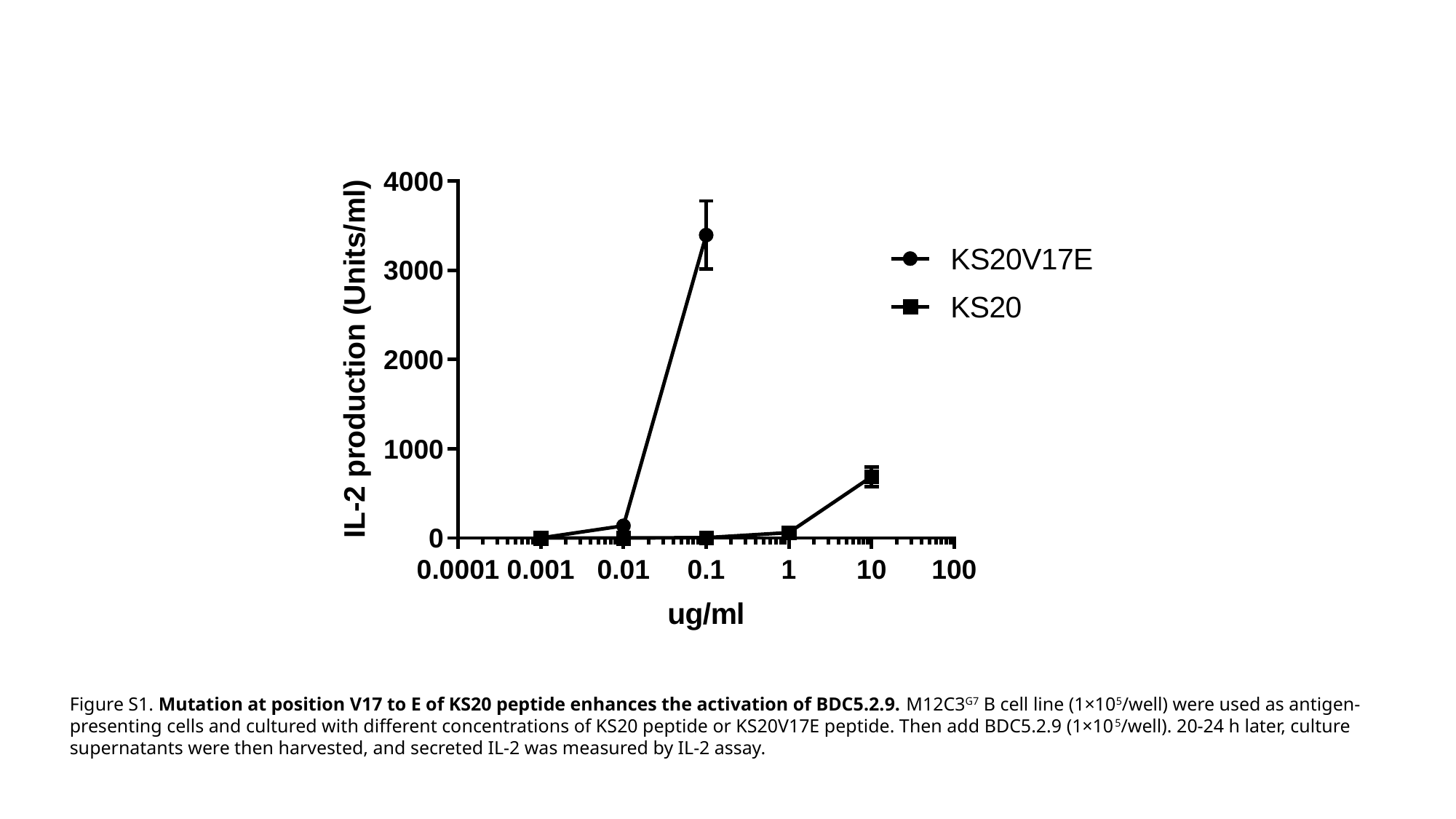

Figure S1. Mutation at position V17 to E of KS20 peptide enhances the activation of BDC5.2.9. M12C3G7 B cell line (1×10^5^/well) were used as antigen-presenting cells and cultured with different concentrations of KS20 peptide or KS20V17E peptide. Then add BDC5.2.9 (1×10^5^/well). 20-24 h later, culture supernatants were then harvested, and secreted IL-2 was measured by IL-2 assay.

## Slide 3
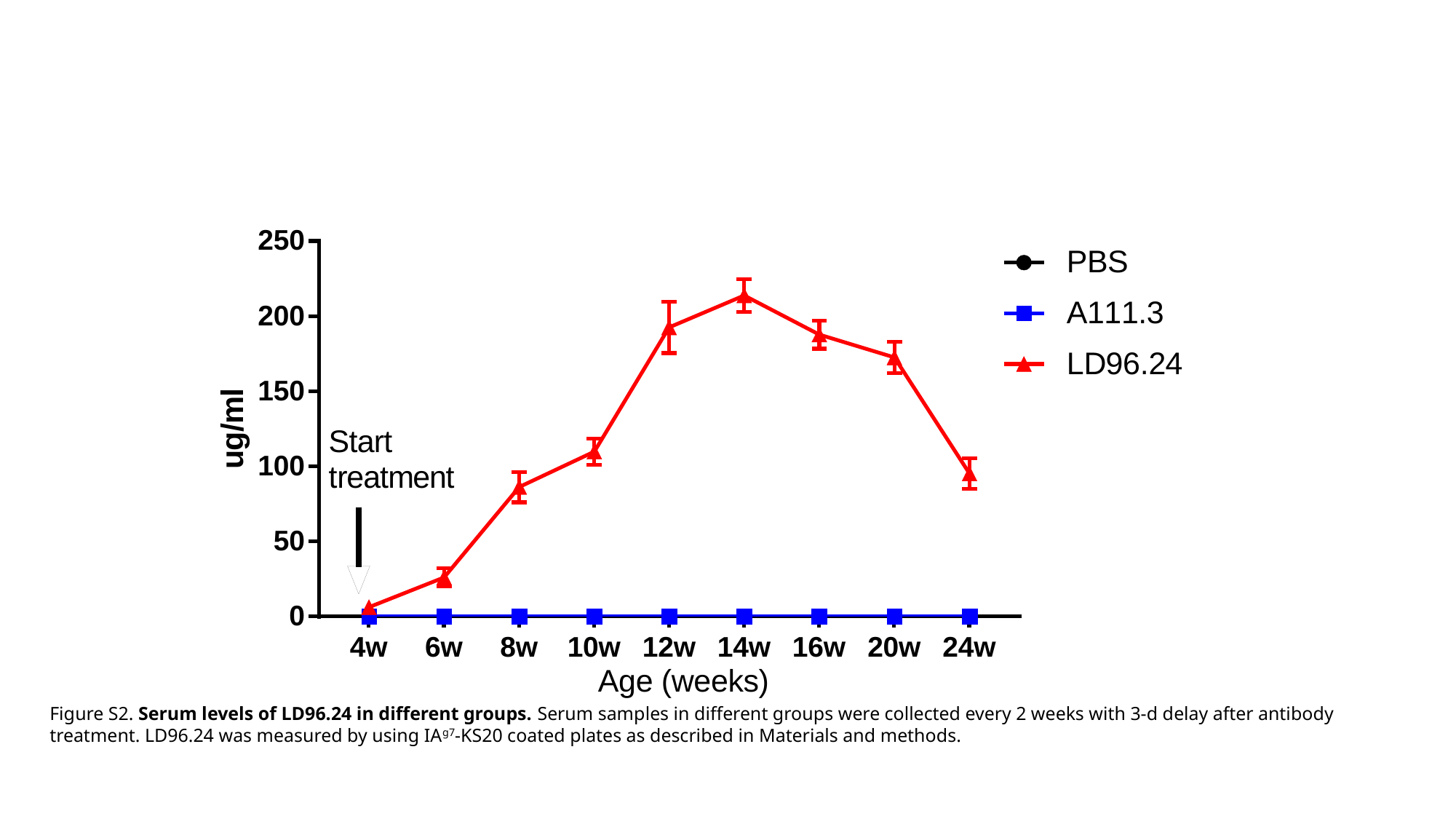

#
Figure S2. Serum levels of LD96.24 in different groups. Serum samples in different groups were collected every 2 weeks with 3-d delay after antibody treatment. LD96.24 was measured by using IAg7-KS20 coated plates as described in Materials and methods.

## Slide 4
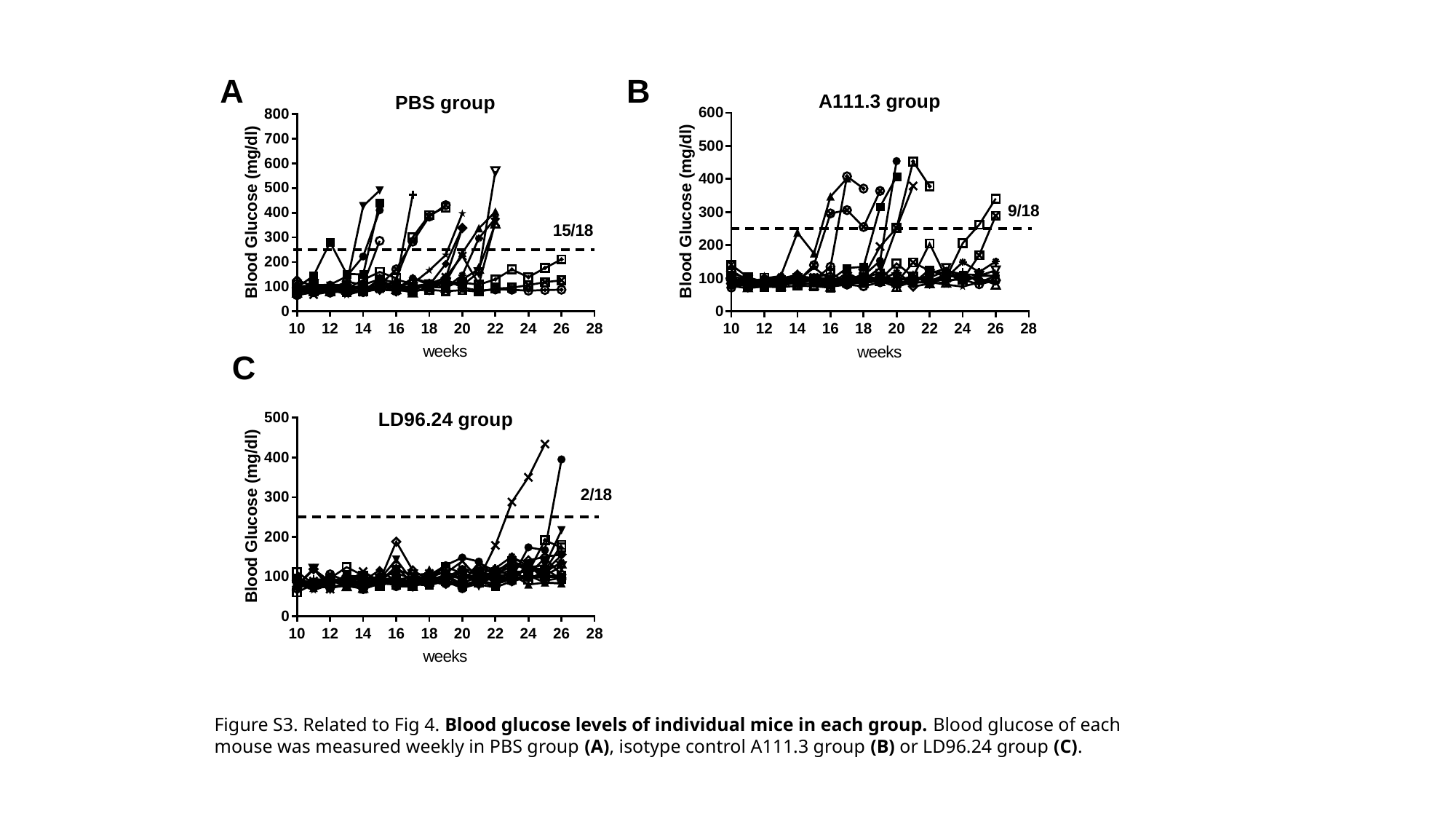

Figure S3. Related to Fig 4. Blood glucose levels of individual mice in each group. Blood glucose of each mouse was measured weekly in PBS group (A), isotype control A111.3 group (B) or LD96.24 group (C).

## Slide 5
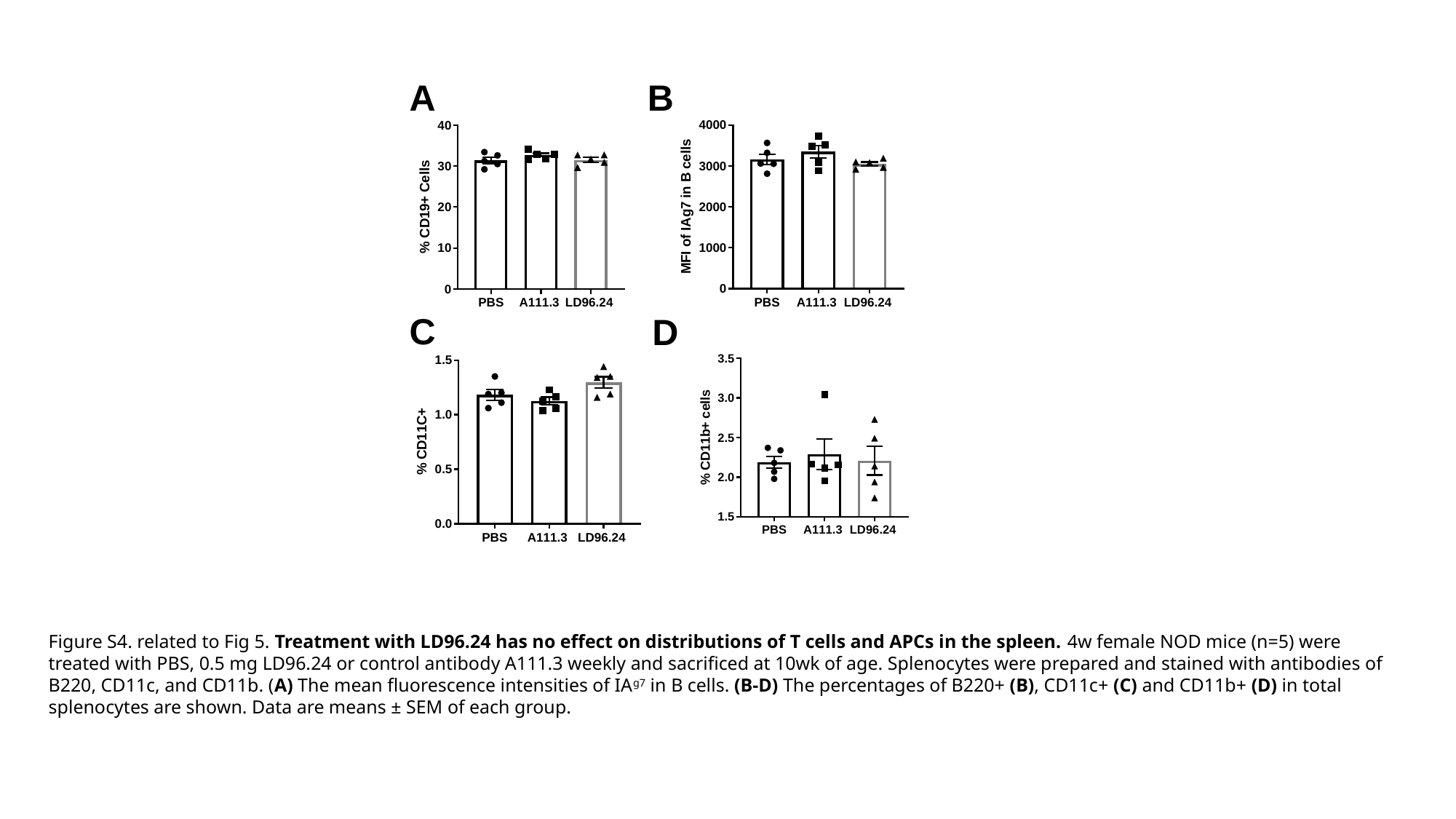

Figure S4. related to Fig 5. Treatment with LD96.24 has no effect on distributions of T cells and APCs in the spleen. 4w female NOD mice (n=5) were treated with PBS, 0.5 mg LD96.24 or control antibody A111.3 weekly and sacrificed at 10wk of age. Splenocytes were prepared and stained with antibodies of B220, CD11c, and CD11b. (A) The mean fluorescence intensities of IAg7 in B cells. (B-D) The percentages of B220+ (B), CD11c+ (C) and CD11b+ (D) in total splenocytes are shown. Data are means ± SEM of each group.

## Slide 6
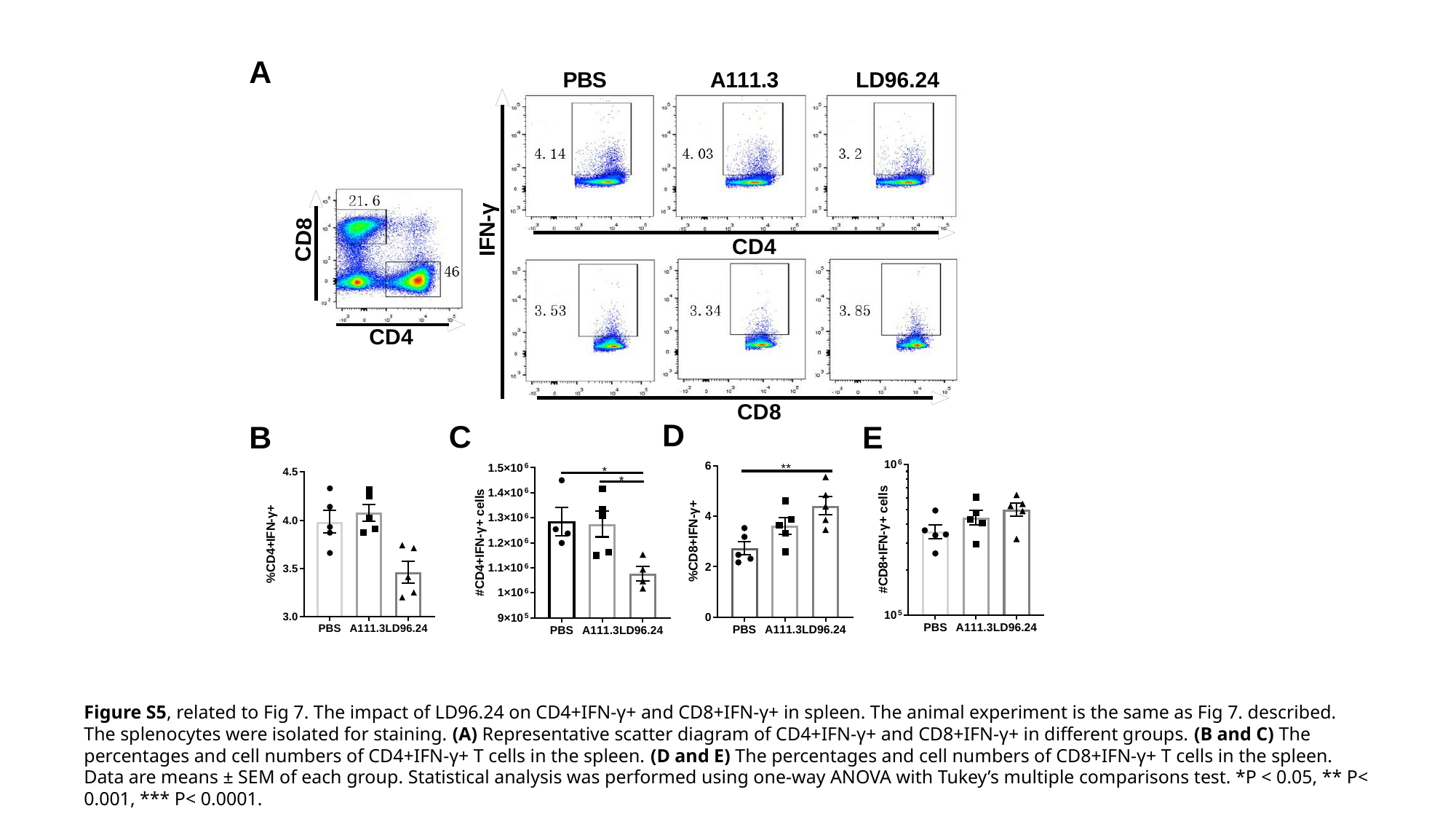

Figure S5, related to Fig 7. The impact of LD96.24 on CD4+IFN-γ+ and CD8+IFN-γ+ in spleen. The animal experiment is the same as Fig 7. described. The splenocytes were isolated for staining. (A) Representative scatter diagram of CD4+IFN-γ+ and CD8+IFN-γ+ in different groups. (B and C) The percentages and cell numbers of CD4+IFN-γ+ T cells in the spleen. (D and E) The percentages and cell numbers of CD8+IFN-γ+ T cells in the spleen. Data are means ± SEM of each group. Statistical analysis was performed using one-way ANOVA with Tukey’s multiple comparisons test. *P < 0.05, ** P< 0.001, *** P< 0.0001.
